# Supplementary material for: Role and mechanism of NCAPD3 in promoting malignant behaviors in gastric cancer
Source: Front Pharmacol. 2024 Apr 22;15:1341039. doi: 10.3389/fphar.2024.1341039 (PMC11070777; doi:10.3389/fphar.2024.1341039)
Supplement: Supplementary file 11 [file DataSheet2.ZIP › GSEA/Transcipt factor/my_analysis.Gsea.1599461814734/NRF1_Q6.html]

Details for gene set NRF1\_Q6[GSEA]

|  || Dataset | filtered\_dataset.sample\_info.cls#WT\_versus\_NCAPD3\_MUT |
| Phenotype | sample\_info.cls#WT\_versus\_NCAPD3\_MUT |
| Upregulated in class | WT |
| GeneSet | NRF1\_Q6 |
| Enrichment Score (ES) | 0.38222826 |
| Normalized Enrichment Score (NES) | 1.7142193 |
| Nominal p-value | 0.029411765 |
| FDR q-value | 0.488613 |
| FWER p-Value | 0.999 |
Table: GSEA Results Summary

  

Fig 1: Enrichment plot: NRF1\_Q6      
 Profile of the Running ES Score & Positions of GeneSet Members on the Rank Ordered List

  

| SYMBOL | TITLE | RANK IN GENE LIST | RANK METRIC SCORE | RUNNING ES | CORE ENRICHMENT || 1 | 51315 | KRCC1 | 0 | 1.527 | 0.1107 | Yes |
| 2 | 5514 | PPP1R10 | 11 | 1.087 | 0.1823 | Yes |
| 3 | 23248 | RPRD2 | 33 | 0.977 | 0.2381 | Yes |
| 4 | 4204 | MECP2 | 70 | 0.856 | 0.2742 | Yes |
| 5 | 146050 | ZSCAN29 | 226 | 0.654 | 0.2099 | Yes |
| 6 | 91574 | C12orf65 | 238 | 0.646 | 0.2489 | Yes |
| 7 | 6426 | SRSF1 | 243 | 0.637 | 0.2922 | Yes |
| 8 | 92806 | CENPBD1 | 249 | 0.631 | 0.3343 | Yes |
| 9 | 163126 | EID2 | 252 | 0.629 | 0.3785 | Yes |
| 10 | 25852 | ARMC8 | 307 | 0.588 | 0.3822 | Yes |
| 11 | 5429 | POLH | 471 | 0.490 | 0.3004 | No |
| 12 | 27229 | TUBGCP4 | 556 | 0.442 | 0.2719 | No |
| 13 | 79053 | ALG8 | 576 | 0.436 | 0.2898 | No |
| 14 | 10445 | MCRS1 | 617 | 0.414 | 0.2910 | No |
| 15 | 57496 | MKL2 | 658 | 0.398 | 0.2910 | No |
| 16 | 9223 | MAGI1 | 737 | 0.357 | 0.2607 | No |
| 17 | 3093 | UBE2K | 795 | 0.326 | 0.2433 | No |
| 18 | 9524 | TECR | 834 | 0.292 | 0.2371 | No |
| 19 | 1984 | EIF5A | 846 | 0.259 | 0.2479 | No |
| 20 | 6397 | SEC14L1 | 941 | -0.364 | 0.2066 | No |
| 21 | 84919 | PPP1R15B | 979 | -0.394 | 0.2085 | No |
| 22 | 22931 | RAB18 | 1231 | -0.608 | 0.0717 | No |
| 23 | 1649 | DDIT3 | 1341 | -0.779 | 0.0497 | No |
Table: GSEA details [plain text format]

  

Fig 2: NRF1\_Q6      
 Blue-Pink O' Gram in the Space of the Analyzed GeneSet

  

Fig 3: NRF1\_Q6: Random ES distribution      
 Gene set null distribution of ES for **NRF1\_Q6**
